# Phenotypic characterization and anticancer capacity of CD8+ cytokine-induced killer cells after antigen-induced expansion

**DOI:** 10.1371/journal.pone.0175704

**Published:** 2017-04-20

**Authors:** Jianhua Liu, Lu Wang, Yaoling Wang, Wenjie Zhang, Yilin Cao

**Affiliations:** Department of Plastic Surgery, Shanghai Ninth People’s Hospital Affiliated to Shanghai Jiaotong University School of Medicine, Shanghai, China; State University of New York, UNITED STATES

## Abstract

Cytokine-induced killer cells (CIK) have been used in clinic for adoptive immunotherapy in a variety of malignant tumors and have improved the prognosis of cancer patients. However, there are individual differences in the CIK cell preparations including the obvious differences in the ratio of effector CIK cells among different cancer patients. Infusion of such heterogeneous immune cell preparation is an important factor that would affect the therapeutic efficacy. We report here the enrichment and expansion of CD8+ cells from CIK cells cultured for one week using magnetic activated cell sorting (MACS). These enriched CD8+ CIK cells expressed T cell marker CD3 and antigen recognition receptor NKG2D. Phenotypic analysis showed that CD8+ CIK cells contained 32.4% of CD3+ CD56+ natural killer (NK)-like T cells, 23.6% of CD45RO+ CD28+, and 50.5% of CD45RA+ CD27+ memory T cells. *In vitro* cytotoxic activity assay demonstrated that the enriched CD8+ CIK cells had significant cytotoxic activity against K562 cells and five ovarian cancer cell lines. Intriguingly, CD8+ CIK cells had strong cytotoxic activity against OVCAR3 cells that has weak binding capability to NKG2D. Flow cytometry and quantitative RT-PCR analysis revealed that OVCAR3 cells expressed HLA-I and OCT4 and Sox2, suggesting that CD8+ CIK cells recognize surface antigen via specific T cell receptor and effectively kill the target cells. The results suggest that transplantation of such *in vitro* enriched and expanded OCT4-specific CD8+ CIK cells may improve the specific immune defense mechanism against cancer stem cells, providing a novel avenue of cancer stem cell targeted immunotherapy for clinical treatment of ovarian cancer.

## Introduction

Cytokine-induced killer cells (CIK) are a type of polyclonal killer T cells that are activated by interferon-gamma (IFN-γ) and CD3 antibody. Since 1990s, CIK cells have been used clinically as adoptive cell therapy for a variety of malignant tumors, and have improved patient outcomes especially in combination with other cancer treatments such as chemotherapy [1–4]. It is generally believed that the anti-cancer effectors in CIK cells are natural killer (NK)-like T cells with CD3+ CD56+ phenotype [5, 6]. These CIK effector cells express NKG2D receptor and recognize cancer cell surface NKG2D ligands (NKG2DL), including MICA, MICB and ULBPs in HLA-unrestricted manner. The binding of NKG2 with NKG2DL promotes the release of perforin and granzyme B leading to subsequent apoptosis of CIK target cells [7–10]. Clinical studies have revealed that CIK cell infusion significantly benefits cancer patients with MICA/B+ expression [11–13]. In addition, CD8+ CIK cells, regardless of CD56 expression, express NKG2D as well as T cell receptor (TCR), and this cell subtype accounts for 60% of total CIK cells [7, 10]. The binding of TCR with HLA-I bound antigen peptides on the surface of target cells is able to transduce signals to generate HLA-restricted function of CIK cells [14, 15]. Therefore, CD8+ CIK cells are believed to be a class of bifunctional cells that have both TCR and NKG2D antigen recognition receptors and are capable of exerting immune killing effects via HLA restricted and unrestricted mechanisms. Clinical data have shown that better therapeutic effects are obtained with CIK cell preparations having higher percentage of CD8+ cells [16]. We accordingly hypothesize that transplantation of CD8+ CIK cells that are sorted from conventional CIK culture and expanded *in vitro* might significantly improve clinical outcomes.

In this study, we enriched CD8+ subsets from cultured CIK cells using magnetic activated cell sorting (MACS) technique, and investigated the proliferation, immune phenotype, antigen recognition mechanism, and ovarian cancer cell killing activity of these CD8+ CIK cells.

## Materials and methods

### PBMC donation volunteers

This study was approved by the Ethics Committee of Shanghai Ninth People's Hospital Affiliated to Shanghai Jiaotong University School of Medicine, and all the volunteers have signed a written informed consent. This study enrolled 31 healthy volunteers including 23 males and 8 females with mean age of 37 years old (range 21–54 years). Inclusion criteria were no history of chronic diseases (such as diabetes, hypertension), viral infections (such as hepatitis), autoimmune diseases (such as systemic lupus erythematosus, rheumatoid arthritis, nephritis) and cancer. The volunteers of cancer patients were advanced epithelial ovarian cancers diagnosed and treated in our hospital.

### Tumor cell lines and culture

3AO, A2780, HO8910, OVCAR3 and SKOV3 ovarian cancer cells and K562 cells were purchased from the Cell Resource Center at Shanghai Institute of Life Science, Chinese Academy of Sciences (Shanghai, China). The cells were cultured in RPMI 1640 medium containing 10% fetal calf serum (FCS, Hyclone), 100 U/ml penicillin and 100 μg/ml streptomycin in an incubator with 5% CO_2_ at 37°C.

### Isolation of Peripheral Blood Mononuclear Cells (PBMC) and culture of CIK cells

Peripheral blood of 20 ml was collected with EDTA anticoagulant from each donor and centrifuged at 400 g for 10 min to remove plasma. The blood cell pellet was resuspended in 20 ml phosphate buffered saline (PBS) and centrifuged at 800 g for 15 in Ficoll centrifuge tube. PBMC at interface cells were collected and resuspended in 40 ml PBS and centrifuged at 400 g for 10 min. The cell pellet was resuspended in 40 ml PBS and centrifuged at 400 g for 10 min for the second time. PBMC was adjusted to 1x10^6^/ml and cultured in 10 ml of GT-T551 culture medium containing 10% FCS, 1000 U/ml IFN-γ (Shanghai Chemo Wanbang Biopharma, Shanghai, China) in T25 flask for 1 day. In the next day, the cells were stimulated with 30 ng/ml of anti-CD3 antibody (T&L Biological Technology, Beijing, China) and 1000 IU/ml of IL2 (Shanghai Huaxin High Biotechnology, Shanghai, China) to induce CIK cell proliferation. The cell culture was supplemented with 1000 IU/ml of IL2 every 1–2 days.

### Magnetic cell sorting (MACS) and CD8+ CIK cell culture

After culture for 1 week, CIK cells were collected by 400 g centrifugation for 10 min and resuspended in 500 μl MACS buffer (Miltenyi Inc), followed by addition of 100 μl CD8 microbeads (Miltenyi Inc.). After incubation at room temperature for 30 min, the cells were mixed with 5 ml PBS, collected by 400 g centrifugation for 10 min, and resuspended in 500 μl PBS. CD8- cells were removed and CD8+ CIK cells were selected using LS column (Miltenyi Inc.) following the manufacturer's operating manual. The final obtained CD8+ CIK cells were resuspended in 5 ml PBS, collected by centrifugation at 400 g for 10 min, and cultured in 10% FCS-GT-T551 culture medium containing 1000 IU/ml IL2 at a density of 1x 10^6^/ml for 2 weeks.

### Immunotyping of CIK cells

5 x 10^5^ CIK cells in 100 μl MACS buffer were stained with fluorescent-conjugated antibodies (BD Biosciences) at 4°C in the dark for 30 min. The cells incubated with MACS buffer alone were used as control. After incubation, 3 ml PBS was added into each tube. The cells were collected by centrifugation at 400 g for 5 min, and resuspended in 500 μl PBS. The positively stained cells were analyzed using Carlibro or C6 flow cytometer (BD Biosciences).

### Degranulation assay

For TCR-induced degranulation assay, 6 x 10^5^ CIK cells in 100 μl PBS were stimulated with 1 μg/ml CD28 antibody (BD Biosciences), 1 μg/ml CMV-pp65 antigen polypeptides (Miltenyi) and stained with 5μμl PE-CD107a antibody (Miltenyi). For NKG2D-induced degranulation assay, 6x10^5^ CD8+ CIK cells were mixed with 6x10^6^ or 6x10^5^ K562 cells in 200 μl, followed by addition of 5 μl PE-CD107a antibody. After incubation at 37°C for 4 hr, the cells were added 3 ml PBS and pelleted by centrifugation at 400 g for 5 min. The cell pellet was resuspended in 100 μl MACS buffer, added with 20 μl FITC-CD8 antibody (BD Biosciences), and incubated at 4°C in the dark for 30 min. Then, the cells were washed once with 3 ml PBS, resuspended in 500 μl PBS, and subjected to flow cytometry analysis of CD8+ CD107a+ cells. The same procedures were performed for OCT4 and Sox2 polypeptide antigens. The sequence of OCT4 peptide was DVVRVWFCNRRQKGK, while that of Sox2 was DYKYRPRRKTKTLMKKDKYTLPG. The antigenic polypeptides used were synthesized by Shanghai Qiangyao Co. Ltd (Shanghai, China), purified by HPLC to purity of 98%, and stored at -80°C with stock concentration of 5 mg/ml.

### Detection of MICA/B and NKG2D-Fc fusion protein

6 x 10^5^ K562 cells in 100 μl MACS buffer were stained with 20 μl mouse anti-MICA/B antibody (BD Biosciences) with the cells in the buffer alone as control and incubated at 4°C in the dark for 30 min. For detection of NKG2D-Fc fusion protein, 6 x 10^5^ ovarian cancer cells in 100 μl MACS buffer were stained with 20 μl recombinant NKG2D-Fc fusion protein (R&D systems). The cells were then respuspended in 3 ml PBS and collected by centrifugation at 400 g for 5 min. The cell pellet was resuspended in 100 μl MACS buffer, added with 5 μl FITC-labeled goat anti-mouse or rabbit IgG (Santa Cruz Company) antibody (BD Biosciences), and incubated at 4°C in the dark for 30 min. Then, the cells were washed once with 3 ml PBS, resuspended in 500 μl PBS, and subjected to flow cytometry analysis.

### Quantitative RT-PCR assay

Total RNA from ovarian cancer and K562 cells was extracted by using RNeasy^R^ Plus Micro kit (Qiagen) according to the manufacturer’s instructions. The quantity and purity of the RNA were assessed by measuring the absorbance at 260 and 280 nm. The cDNA was synthesized from total RNA (2 μg) with oligo (dT) primers using a QuantiTect Reverse transcription Kit (Qiagen). All PCR conditions were optimized to produce a single product in the exponential range. Quantitative real-time PCR was performed using HotStar Taq^™^Plus DNA kit and analyzed using an ABI Step One real time PCR System (Applied Biosystems, Foster City, CA, USA). Primer sequences for the reference gene 18S rRNA and the genes of interest are listed in Table 1. Typical PCR thermocycler profile was the initial step at 95°C for 10 min followed by a second step at 95°C for 15 s and 60°C for 30 s for 40 cycles with a melting curve analysis. The level of target mRNA was normalized to the level of the 18S rRNA and compared with the control. Data were analyzed using the ^ΔΔ^CT method.

**Table 1:** PCR primer sequences.

GENE	Forward (5’ - 3’)	Reverse (5’ -3’)
18S rRNA	TCGGAGGTTCGAAGACGATC	CAGCTTTGCAACCATACTCCC
MICA	ACGGCGATATCTAAAATCCGG	TGACGCCAGCTCAGTGTGAT
MICB	GACCAAGACACACTATCGCGC	CATGTCACGGTGATGTTGCC
ULBP1	TTAAAGGGCAACTGCTTGACATT	AGAGGAGGAACTTCTGTCCATTGA
ULBP2	GAGGTGGTGGACATACTTACAGAGC	CCCATCGAAACTGAACTGCC
ULBP3	AGGCTCAGACTGGAACTGGC	GAGGAACTTCCGTCCATCGA
ULBP4	CAATGCCAGAACCGACAGTGT	CACCATTTTGCCACCAGACA
OCT4	GGGTGGAGAGCAACTCCG A	GCTTGGCAAATTGCTCGA G
Sox2	GTTCTAGTGGTACGGTAGGAGCTTTG	TTTGATTGCCATGTTTATCTCGAT

### Analysis of HLA-I molecules expression

6x 10^5^ K562 cells or ovarian cancer cells in 100 μl MACS buffer were added with 20 μl FITC-HLA-ABC antibody (BD Biosciences) and incubated at 4°C in the dark for 30 min. The cells were then resuspended in 3 ml PBS, collected by centrifugation at 400 g for 5 min, and resuspended in 500 μl PBS. The HLA+ cells were analyzed using Carlibro or C6 flow cytometer (BD Biosciences).

### Cytotoxic activity assay

Target cells (K562 and ovarian cancer cells) in 2 ml PBS, were labeled with 2 μM fluorescent dye CFSE (Sigma) at room temperature for 5 min and terminated by addition of 1 ml FCS. The fluorescence labeled cells were washed with 20 ml PBS by centrifugation (400 g, 10 min) for 3 times, resuspended in 10% FCS-RPMI medium, and cell density was adjusted to 1x 10^6^/ml. Effector cells (CIK or CD8+ CIK cells) were collected, resuspended in 10% FCS-RPMI medium, and cell density was adjusted to 1x 10^7^/ml and 2x 10^7^/ml. In experimental group, effector cells and target cells were mixed in equal volumes (100 μl each) to give the ratio of effector/target cells (E/T) of 10: 1 and 20: 1. The spontaneous death group was target cells only. The cell mixture were centrifuged at 400 g for 5 min and cultured at 37°C for 4 hr, followed by addition of 10 μl DNA fluorescent dye PI (100 mg/ml, Sigma) and incubation for 15 min. The cells were then washed once with 3 ml PBS, resuspended in 500 μl PBS and subjected to flow cytometry analysis. Specific cytotoxicity was calculated according to the formula: % specific killing = [1] x 100%.

## Results

### MACS sorting of in vitro expanded CIK cells results in high homogeneity of CD3+CD8+ cells expressing NKG2D antigen receptors

**Fig 1:**
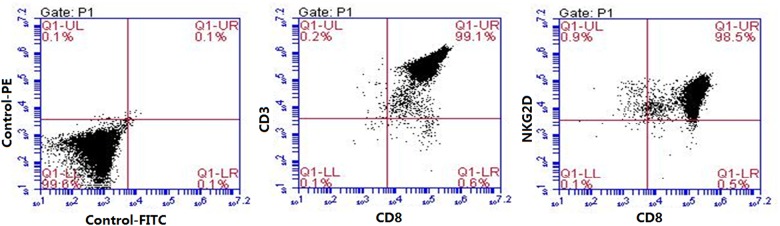
Analysis of CD8 + cell purity by flow cytometry. CD8+ CIK cells were labeled with anti-CD8/CD3 and CD8/NKG2D specific fluorescent antibody and subjected to flow cytometry. The control group (control) was treated with PBS instead of antibody. Shown are representative images of cells from Donor 5.

The PBMC from 17 healthy volunteers were used to assess the purity and proliferation of CD8+ CIK cells. After stimulation by IFN-γ and CD3 antibody for 7 days, the average of PBMC number increased 3.46 times reaching to a mean of 1.35 x 10^8^. After MACS sorting, the rate of CD8+ CIK cell subsets was 34.8% with a mean cell count of 0.47 x 10^8^ (Table 2). Further culture of the sorted CD8+ CIK cells with 1000 IU/ml IL2 for 2 weeks led to the increase of the cell number of 91.7 times reaching 43.1 x 10^8^/ml. Cell purity analysis of the expanded CD8+ CIK cells demonstrated an average of 95.1% of CD3+ CD8+ cells and 93.4% of CD8+ NKG2D+ cells (Table 2). Fig 1 showed the representative results of flow cytometry (D5 donor cells). Thus, after sorting through MACS, high homogeneity of CD3+CD8+ cells expressing NKG2D antigen receptors were obtained.

**Table 2:** The cell purity and proliferation ability of CIK and CD8+ CIK cells.

PBMC donor ID	CIK culture			CD8^+^ CIK culture			CD8^+^ cell purity (%)	
	PBMC N	Culture days	CIK N	CD8^+^ N	Culture days	Total N	CD3^+^CD8^+^	CD8^+^NKG2D^+^
D1	0.34	5	1.29	0.57	17	8.60	98.8	97.6
D2	0.28	6	1.22	0.61	16	10.74	90.4	95.5
D3	0.2	7	1.14	0.34	17	1.31	98.5	97.6
D4	0.58	6	1.58	0.63	18	4.56	93.3	95.5
D5	0.36	6	1.31	0.55	18	8.10	99.1	98.5
D6	0.32	6	1.29	0.51	17	4.52	98.4	95.5
D7	0.32	7	1.21	0.46	14	2.87	99.2	99.3
D8	0.23	7	1.13	0.36	14	3.11	95.4	97.9
D9	0.96	7	2.25	0.47	18	4.93	85.7	84.5
D10	0.46	7	1.33	0.32	16	1.28	98.8	97.0
D11	0.4	7	1.22	0.31	11	0.93	93.1	95.4
D12	0.24	8	1.07	0.32	10	1.32	99.2	94.4
D13	0.36	8	1.31	0.52	10	5.59	97.9	94.6
D14	0.52	8	1.55	0.47	10	4.12	95.3	93.2
D15	0.38	8	1.37	0.55	12	4.99	93.2	91.6
D16	0.36	7	1.42	0.54	14	3.72	97.0	91.1
D17	0.4	7	1.30	0.39	14	2.61	83.4	68.9
Mean	0.39	6.88	1.35	0.47	14.47	4.31	95.1	93.4

Note: PBMC number: the number of 40 ml of peripheral blood cells after Ficoll centrifugation and used for CIK cell culture (x 10^8^/ml); CIK cell number: the number of CD8+ cells after MACS sorting of CIK cells (x 10^8^/ml); CD8+ cell number: the number of CD8+ cells after MACS sorting of expanded CIK cells (x 10^8^/ml); Total cell number: the number of total cells of CD8+ CIK cell when cell expansion culture was terminated (x 10^9^/ml). D1 to D17, the identification (ID) number of the donors. N, the number of cells.

### Immune phenotypes of CD8+ CIK cells

We next analyzed the immune phenotypes of CD8+ CIK cells of 8 cases of expanded cultures of CD8+ CIK cells. Consistent with that of the above 17 cases, these eight cases of CD8+ CIK cell culture contained an average of 96.8% CD3+ CD8+ cells and 94.8% CD8+ NKG2D+ cells. Moreover, the average cell population of CD3+ CD56+ cells was 32.4%. In addition, the average cell populations of CD45RO+ CD28+ and CD45RA+ CD27+ memory T cell phenotype were 23.6% and 50.5%, respectively (Table 3).

**Table 3:** The phenotypes of 8 cases of CD8 + CIK cell after expansion.

CIK ID	Cell purity		Cell phenotype		
	CD3^+^CD8^+^%	CD8^+^NKG2D^+^%	CD3^+^CD56^+^%	CD454RO^+^CD28^+^%	CD45RA^+^CD27^+^%
D18	99.2	98.0	34.3	28.9	52.1
D19	94.1	93.5	30.4	11.3	49.6
D20	97.0	96.5	32.8	24.6	51.9
D21	98.0	96.5	31.9	22.1	51.7
D22	98.4	97.8	33.3	18.1	49.1
D23	93.2	91.6	32.0	24.4	49.3
D24	98.0	88.7	28.9	28.7	49.1
D25	96.1	95.8	35.2	30.5	50.8
Mean	96.8	94.8	32.4	23.6	50.5

Note: D18 to D25, the identification (ID) number of the donors.

### TCR on CD8+ CIK cell surface is functional and activation of it by antigen is capable of selectively increasing the ratio of antigen specific T cells

**Fig 2:**
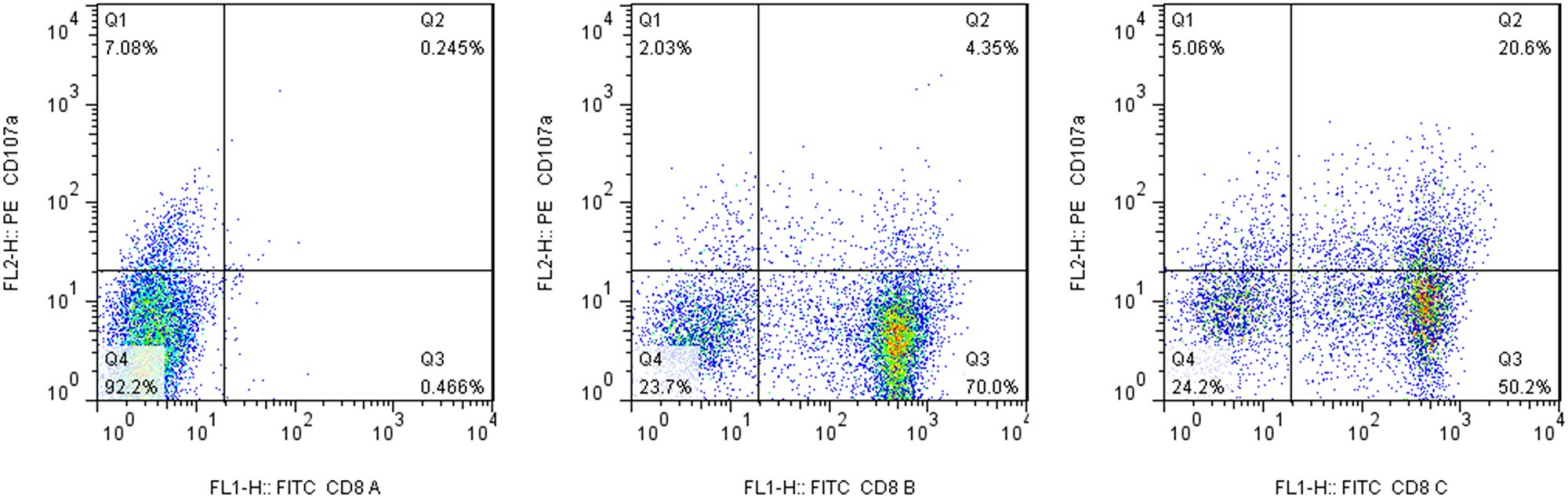
CMV antigen induced PBMC degranulation. Left: control group with no stimulation and fluorescence-labeled antibody addition; Middle: CD28 antibody stimulation alone; Right: CD28 antibody and CMV-pp65 antigen costimulation.

Activated CD8+ T cells by specific antigen obtain immune memory and undergo rapid activation and degranulation to release perforin and granzyme from lysosomes upon confrontation with the same antigen. Meanwhile, the degranulation leads to lysosomal membrane fusion with the cell membrane resulting in cell surface expression of lysosomal membrane protein CD107a. Therefore, the detection of CD107a expression on the surface of CD8+ T cells has been widely used to assess the killing capability of cytotoxic T lymphocyte (CTL). Moreover, antigen activates CD8+ memory T cells through TCR recognition mechanism, which requires costimulatory signals of a complex formed by antigen polypeptide and cell surface HLA molecule and CD28-B7 (CD80/86). In this study, we treated the PBMC from two healthy volunteers with CD28 antibody alone or in combination with CMV-pp65 polypeptide antigen for 4 h. There were very few CD8+ CD107a+ cells in PBMC treated with CD28 antibody alone, whereas the CD8+ CD107a+ cells in the PBMC treated CD28 antibody in combination with CMV-pp65 polypeptide antigen from two cases were 3.12% and 4.51% (Fig 2), indication of antigenic specificity activation of CD107a.

**Fig 3:**
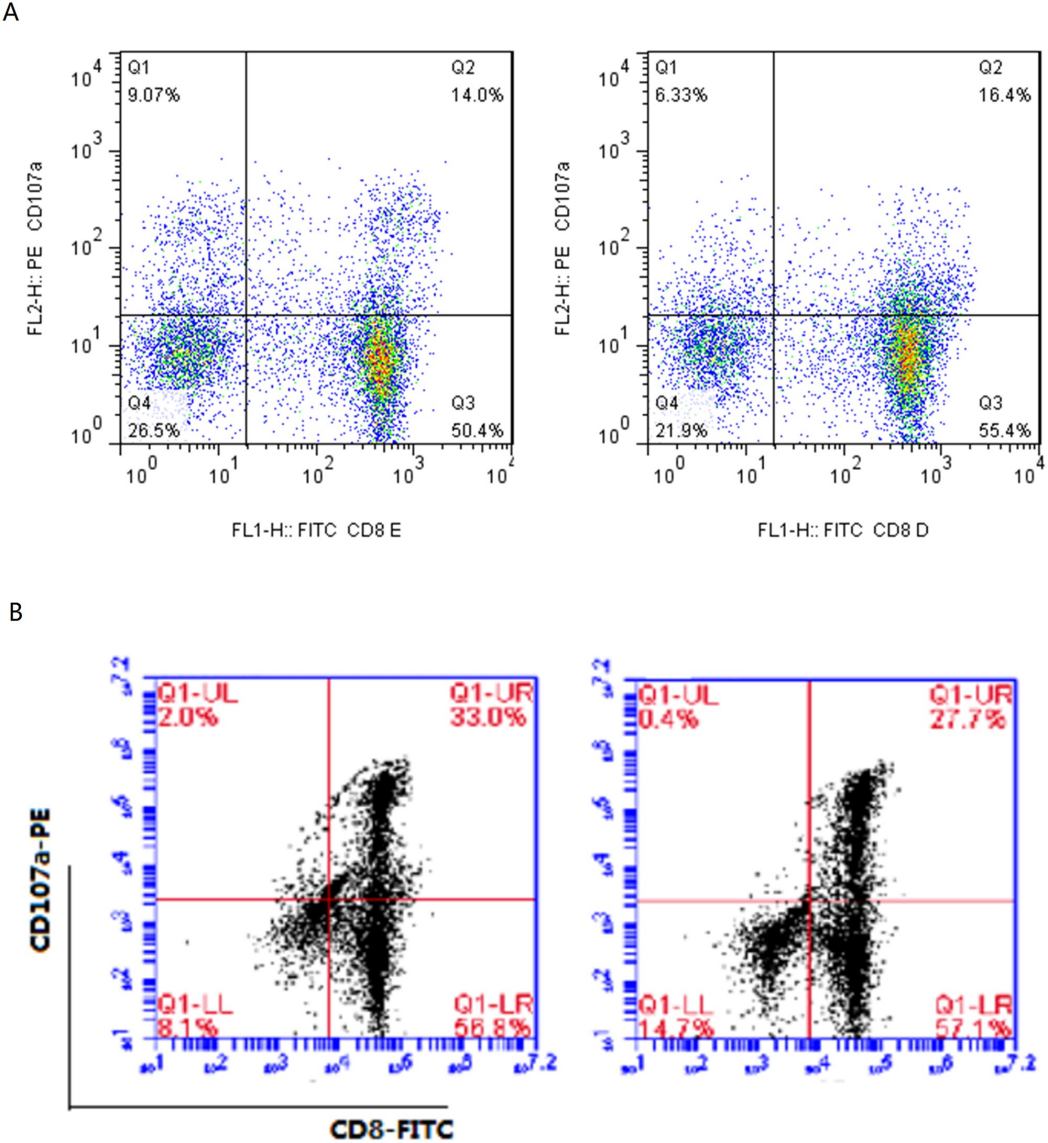
OCT4 and Sox2 antigens induced CD8+ T cell degranulation. A, PBMC were treated with OCT4 and Sox2 polypeptide antigen for 4 h and subjected to flow cytometry analysis of CD8+ CD107a+ cells. B, CIK cells were stimulated with 10 g/ml OCT4 or Sox2 antigen 48 hr prior to MACS sorting, and the sorted CD8+ CIK cell were cultured for additional 2 weeks, followed by flow cytometry analysis of CD8+ CD107a+ cells. left: OCT4 antigen stimulation; Right: Sox2 antigen stimulation.

To expand our observation, we treated the PBMC from four healthy volunteers with OCT4 and Sox2 polypeptide antigens for 4 h and detected average of 3.3% (range 1.1%–5.2%) CD8+ CD107a+ cells (Fig 3A), indicating that CD8+ T cells in healthy adults have immunological memory capacity to OCT4 and Sox2, polypeptide antigens of embryonic stem cells. To test the effect of these antigens on the activation of CD8+ CIK cells, CIK cells were stimulated with 10 μg/ml OCT4 or Sox2 antigen 48 hr prior to MACS sorting, and the sorted CD8+ CIK cell were cultured for additional 2 weeks. Our results showed that the CD8+ CD107a+ cells treated with OCT4 and Sox2 antigen were 33.0% and 27.7%, respectively (Fig 3B). Further analysis of CD8+ CIK cells from 5 additional healthy volunteers showed that the average of CD8+ CD107a+ cells treated with OCT4 was 25.3% (range 21.2%–35.3%), and that of Sox2 was 21.1% (range 18.5%–29.7%). These results suggest TCR on CD8+ CIK cell surface is functional and activation of it by antigen is capable of selectively increasing the ratio of antigen specific T cells.

### NKG2D on CD8+ CIK cell surface is functional and can be activated by MICA/B antigen positive K562 cells

**Fig 4:**
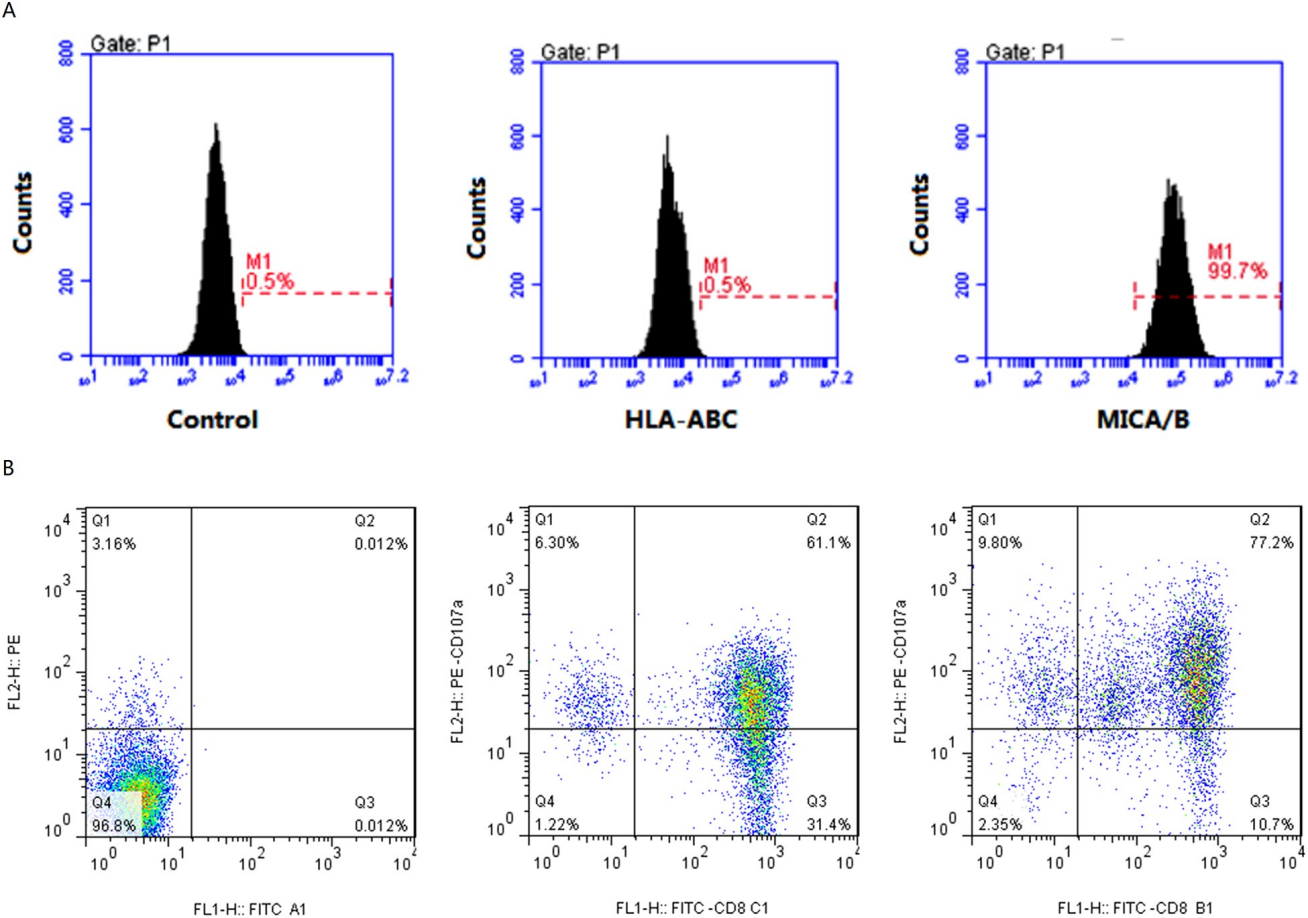
K562 cells induce CD8 + CIK cell degranulation. A, Detection of HLA-ABC and MICA/B antigen expression on K562 cell surface by flow cytometry. B, CD8+ CIK cells were mixed with K562 cells were cultured 4 hr and subjected flow cytometry analysis of CD107a expression. Left: control group with no stimulation and fluorescence-labeled antibody addition; Middle: CD8+ CD107a+ cell The rate of CD8+ CIK cell to K562 cells was 1: 1. Right: CD8+ CD107a+ cell The rate of CD8+ CIK cell to K562 cells was 10: 1.

To test NKG2D receptor function on CD8+ CIK cell surface, we chose K562 cells as stimulator cells. We first examined the HLA expression on K562 cell surface and found no undetectable HLA molecules on K562 cell surface (Fig 4A). We then detected MICA/B antigen expression on K562 cell surface and found that MICA/B was positive in 99.7% of the cells. Accordingly, we co-cultured CD8+ CIK cells and K562 cells with different ratio for 4 h, and discovered that CD8+ CD107a+ cell rates with 10: 1 and 1:1 of CD8+ CIK cell to K562 cells were 77.2% and 61.1% (Fig 4B), respectively, implying activation of CD8+ CIK cells by K562 cells.

### Ovarian cancer cells express distinct NKG2D ligands at different levels

To assess the cytotoxic activity of CD8+ CIK cells on ovarian cancer cells, we screened a panel of ovarian cancer cell lines for the expression of NKG2D ligands including MICA, MICB and ULBP1-4 by qPCR. By using of K562 cells as a reference (Table 4), we found that MICA gene expression in the five ovarian cancer cell lines was significantly lower than K562 cells, while MICB and ULBP2 expression levels were similar to that in K562 cells; there was increased expression of ULBP3 in HO8910, OVCAR3 and SKOV3 cell; Increased ULBP4 expression was observed in 3AO, A2780 and HO8910 cells, whereas ULBP1 showed a low expression in all ovarian cancer cells with undetectable ULBP1 in A2780, OVCAR3 and SKOV3 cells. These results showed that these ovarian cancer cells expressed different NKG2D ligands at different levels.

**Table 4:** Gene expressions of NKG2D ligands in ovarian cancer cell lines.

	MICA	MICB	ULBP1	ULBP2	ULBP3	ULBP4
K562	1	1	1	1	1	1
3AO	0.04	0.69	0.15	3.86	3.32	17.27
A2780	ND	3.23	ND	ND	ND	53.82
HO8910	0.04	0.73	0.10	1.95	11.96	10.63
OVCAR3	0.06	0.21	ND	0.76	18.51	1.46
SKOV3	0.95	0.97	ND	2.45	59.30	2.50

ND: not detectable. The data shown are 2^-Ct^ relative to 18S rRNA compared with that of K562 cells.

**Fig 5:**
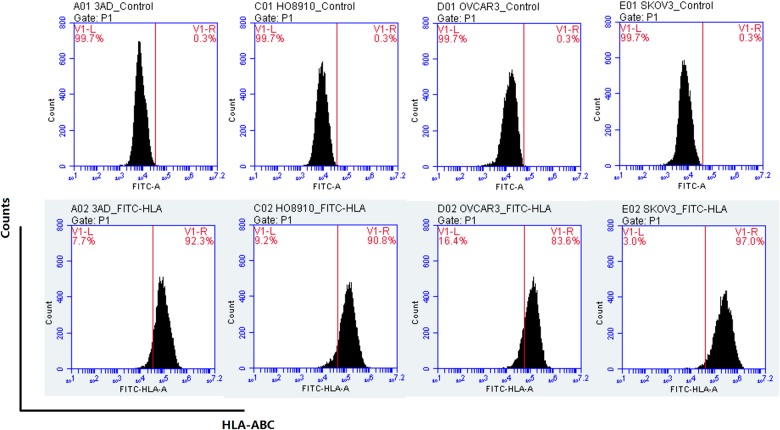
Detection of the binding capability of ovarian cancer cell surface NKG2DL with NKG2D receptor with NKG2D-Fc recombinant protein. The upper panel shows the control without addition of NKG2D-Fc recombinant protein.

We next used recombinant NKG2D-Fc fusion protein to detect binding capacity of ovarian cancer cell surface NKG2DL, and found the positive NKG2D staining rates in OVCAR3, SKOV3, A2780, HO8910 and 3AO cells were 8.2%, 10.5%, 42.1%, 67.8% and 75.1%, respectively (Fig 5). In combination with the expression status of NKG2D ligands (Table 4), these results suggest ULBP4 may be the main cell surface NKG2D ligand for NKG2D receptor in ovarian cancer cells.

### CD8+ CIK cells have a stronger cell killing activity against K562 cells than CIK cells

**Fig 6:**
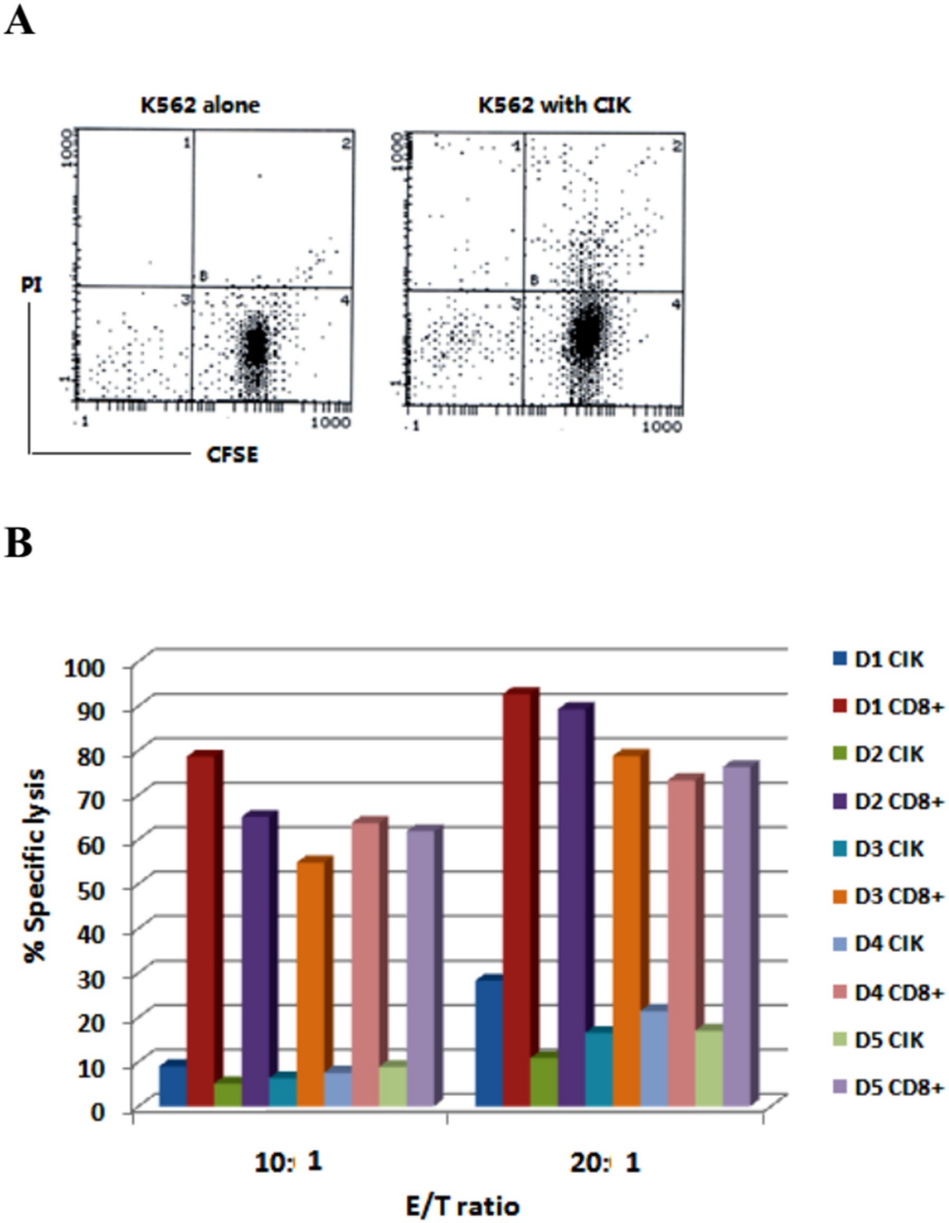
The cell killing activity of CIK cells and CD8+ CIK cells against K562 target as analyzed by flow cytometry. A. K562 cells were cultured alone or co-cultured with CIK cells (1:10) for 4h. Cell death was analyzed with PI and CFSE staining coupled with flow cytometry. B. K562 cells were co-cultured with CIK or CD8+ CIK cells from five donors (D1-D5) at ration of 1:10 or 1:20 for 4h. Cell death was analyzed with PI and CFSE staining coupled with flow cytometry.

To assess the killing activity of CD8+ CIK cells on target cells, K562 cells were labeled with red fluorescent dye CFSE and mixed with CIK cells. After culture for 4 hr, the cells were added with PI to detect dead cells by flow cytometry (Fig 6A). Using this method, we assessed K562 cell killing activity of five cases of CIK and CD8+ CIK cells. The results showed that, at E/T ratio of 10: 1 and 20: 1, the average killing activity of CIK cells against K562 cells were 7.38% and 19.79%, while the average killing activity of CD8+ CIK cells against K562 cells were 64.79% and 82.07%, respectively (Fig 6B), indicating CD8+ CIK cells have a stronger anti-cancer function than CIK cells (p<0.05).

### CD8+ CIK cells have a stronger cell killing activity against ovarian cancer cells than CIK cells

**Fig 7:**
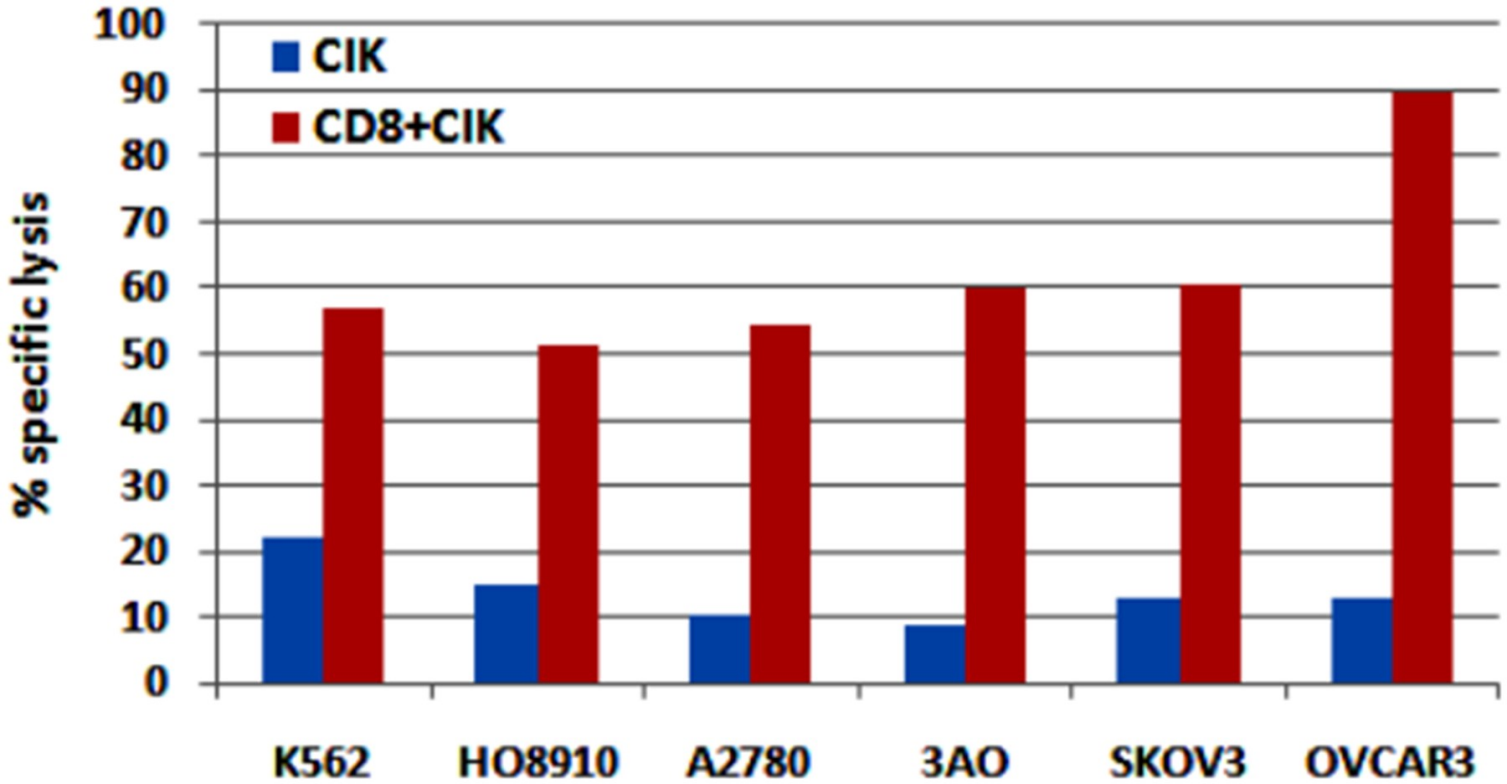
The cell killing activity of CIK cells and CD8+ CIK cells against ovarian cancer cells as analyzed by flow cytometry. The indicated ovarian cancer cells co-cultured with CIK cells or CD8+ CIK cells (1:10) for 4h. Cell death was analyzed with PI and CFSE staining coupled with flow cytometry.

To assess the killing activity of CD8+ CIK cells on ovarian cancer cells, we prepared CIK cells and CD8 + CIK cells from PBMC of two donors and tested their cytotoxic activity in ovarian cancer cells E/T = 10: 1. As shown in Fig 7, the killing ability of CIK cells from one donor against ovarian cancer cells was weaker than K562 cells with the killing activity of less than 15%, while CD8+ CIK cell killing activity against ovarian cancer cells were more than 50%, with the strongest effect on OVCAR3 cells (Fig 7). Similar results were obtained for CIK cells and CD8 + CIK cells from the other donor (data not shown). These results demonstrated that CD8+ CIK cells have a stronger anti-ovarian cancer function than CIK cells.

### Ovarian cancer cells express HLA-I molecules and SOX2 and OCT4

**Fig 8:**
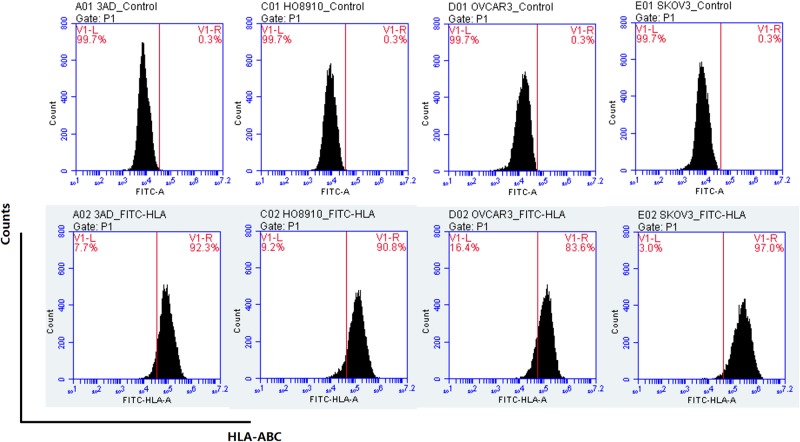
Detection of the expression of HLA-I molecules on ovarian cancer cell surface by HLA-ABC antibody. The upper panel shows the control without addition of HLA-ABC antibody.

The above results indicated that the cell killing activity of the prepared CD8+ CIK cells might be partially independent on the NKG2D antigen-binding on ovarian cancer cells, particularly OVCAR3 cells which had weak NKG2D binding capacity but 80% were killed by CD8+ CIK cells suggesting the existence of other antigen recognition mechanisms by CD8+ CIK cells. To test this hypothesis, we first assessed ovarian cancer cell surface HLA-I molecules using HLA-ABC antibody and found the positive rates of HLA-I molecules in OVCAR3, HO8910, 3AO and SKOV3 were 83.6%, 90.8%, 92.9% and 97.0%, respectively (Fig 8).

Our results indicated that CD8+ T cells in healthy adults might have immunological memory capacity to OCT4 and Sox2 (Fig 3A), suggesting that CD8+ CIK cells may kill ovarian cancer cells via binding OCT4 and Sox2 antigens. To this end, we determined the gene expression of Sox2 and OCT4 in ovarian cancer cells by qPCR. OCT4 mRNA was significantly upregulated in all tested cell lines with relatively lower expression in HO8910 cells (all p<0.01); Sox2 was highly expressed in OVCAR3 but not detectable in the rest cell lines (P<0.01) (Table 5). Taken together, these results demonstrated that that ovarian cancer cells expressed HLA-I molecules and OCT4, and OVCAR3 cells expressed Sox2. Since OCT4 and Sox2 are markers of cancer stem cells, it is possible CD8+ CIK cells exert anticancer effects through specific TCR to recognize cell surface HLA-antigen peptide complex.

**Table 5:** OCT4 and Sox2 gene expression in ovarian cancer cell lines.

Ovarian cell lines	OCT4 expression	P value	Sox2 expression	P value
HO8910	1	<0.01	ND	
3AO	2.53	<0.01	ND	
A2780	56.89	<0.01	ND	
OVCAR3	7.16	<0.01	407.31	<0.01
SKOV3	9.38	<0.01	ND	

ND: not detectable. The data shown are 2^-Ct^ relative to 18S rRNA compared with that of HO8910 cells.

## Discussion

CIK cell transfusion is being widely used for adoptive cell therapy, but the quality of CIK cell preparations is unstable. For example, there are obvious differences in the content of effector cells including CD3+ CD56+ and CD3+ CD8+ cells in the CIK cells from different patients, which affect the therapy efficacy [11–13]. In this study, we enriched CD8+ subsets from cultured CIK cells using MACS technique and obtained high homogeneity of CD8+ CIK cells. Moreover, we demonstrated that CD8+ CIK cells could be activated by HLA-MICA/B through NKG2D receptor as well as by OCT4 and Sox2 antigen via TCR, suggesting CD8+ CIK cells are a class of bifunctional effector cells. Regarding that OCT4 and Sox2 are specific markers cancer stem cells (CSC) [17, 18], we for the first time revealed that CD8+ CIK cells are able to recognize CSC specific antigens.

Establishment of long-term immune defense mechanism in the body after T cells infusion is the key to clinical efficacy. In the T-cell population, only memory T cells have this feature and express CD28 and CD27 costimulatory molecules and other receptors. CD28 belongs to the immunoglobulin (Ig) receptor superfamily and is the specific surface receptor of B7 costimulatory molecules CD80 and CD86. When receiving specific epitopes by antigen presenting cells (APC) surface HLA, the corresponding TCR recognition of the signal (the first signal) and interaction with CD3 and CD28 leads to T cell activation. CD28-B7 interaction provides a second signal for T cell activation. CD27 belongs to the TNF receptor family (also known as TNFRSF7). Interaction of CD27 with APC surface CD70 (TNFSF7) results in recruitment of adaptor proteins TRAF2/5 and activation of JNK kinase and NF-κB signaling cascade, thereby promoting proliferation and inhibiting apoptosis of T cells, indication of an important role for CD27-CD70 signal in the proliferation of activated T cells especially CD8+ T cells [19–22]. In addition to CD27 and CD28 receptors, memory T cells also express other surface markers such as CD45RA and CD45RO. CD45 is a protein tyrosine phosphatase and encoded by *CD45* gene containing seven exons. The encoded full-length product is CD45RA, which is expressed on naive T cell surface together with antigen CD27 and CD28. When naive T cells are activated, the exons 4–6 of CD45 transcript are clipped, producing a short-chain product CD45RO, which is selectively expressed in the memory T cells. When the memory T cells proliferate and differentiate into effector T cells, CD45RA is re-expressed. At the same time, CD45RO is inhibited. Such effector T cells do not express CD45RO, CD27 and CD28 [23]. However, some studies have shown that under appropriate culture conditions, memory T cells especially CD27 + subsets can also express CD45RA [24]. However, it remains unclear whether CD8+ CIK cells have the phenotype and function of memory T cells. Linn et al., reported that CD3+ CD56+ CIK cells are T cells of a late stage with similar phenotypic characteristics to effector memory T cells, such as containing 60% CD28-CD27+ cells, and one-third CD3+ CD56- CIK cells have central memory CD28+ CD27+ T cell phenotype [25]. Nevertheless, another study showed that CD3+ CD56+ CIK cells are derived from CD3+ CD8+ T cells and express CD27 and CD45RA [5]. In our study, using CD27, CD28, CD45RA and CD45RO specific antibodies, we tested the phenotypic characteristics of the sorted and expanded homogeneous CD8+ CIK cell population and found that about 50% of the cells were CD45RA+ CD27+, 23.6% were CD45RO+ CD28+, and 32.4% were CD3+ CD56+ cells. Our results suggest that CD8+ CIK cells have memory T cell phenotype.

It is now believed that one of the anticancer mechanisms of CIK cells is through NKG2D cell surface receptor to recognize the corresponding NKG2DL leading to degranulation and consequent apoptosis of target cells [25]. This effect is not limited by HLA. CD8+ CIK cells express NKG2D and can be activated in the event of HLA-MICA/B + cancer cells such as K562 cells leading to expression of CD107a. Verneris et al. reported that CD8+ cells activated by IFNγ and CD3 antibody express NKG2D, and target NKG2DL+ cells with high cytotoxic activity [10]. Early studies have shown that the expression rate of MICA/B and ULBP1-4 is 60–88% in ovarian cancer tissues, while expression of MICA/B and ULBP2-4 is more common in ovarian cancer cell lines [26, 27]. Using qPCR and NKG2D-Fc fusion protein detection assay, we found ULBP4 might be the main NKG2D receptor recognizing ligand. Moreover, we showed that purified CD8+ NKG2D+ CIK cells effectively killed both K562 and ULBP4+ ovarian cancer cells. Regarding high expression of ULBP4 predicts poor survival of ovarian cancer patients [7], our findings suggest that such CIK cells have potential clinical value in ovarian cancer immunotherapy.

Importantly, by *in vitro* cytotoxic activity assay, we found that the above CIK cells had strong killing activity of OVCAR3 cells that had weak NKG2D binding. Further investigation revealed that these CIK cells might also recognize OCT4 and Sox2 polypeptide antigen via HLA-TCR. OCT4 and Sox2 are specific markers of pluripotent cells (such as embryonic stem cells, ESC) during early embryonic development. OCT4 and Sox2 are necessary transcription factors in maintenance of the self-renewal of stem cells [28, 29]. It has now been widely accepted that CSC are the origin of tumorigenesis and drug resistance, and thus elimination of CSC may be the key to cure cancers [30]. Samardzija et al. observed the expression of OCT4 in ovarian CSC [31], and Di et al. found that peripheral blood T cells from ovarian cancer patient could be activated by OCT4 and became CD107a+ cells [32]. However, there has been no report about the scale preparation of these OCT4 antigen-specific T cells. Our results showed that sorted and expanded CD8+ CIK cells were activated by ovarian cells expressing OCT4 and Sox2, suggesting these CD8+ CIK cells may effectively target CSC of ovarian as well as other cancers.

In summary, CD8+ cells cultured for one week were successfully enriched by MACS and expanded. These enriched and expanded CD8+ CIK cells from CIK had significant cytotoxic activity against K562 and ovarian cancer cells, which expressed HLA-I and OCT4 and Sox2. The results suggest that tranplantation of such *in vitro* enriched and expanded OCT4-specific CD8+ CIK cells may improve the specific immune defense mechanism against CSC, providing a novel avenue of CSC targeted immunotherapy for clinical treatment of ovarian cancer.

## Supporting information

S1 File Data. The raw data for drawing the Figs 6 and 7.
(XLSX)
